# Effect of treatment with phosphate, casein phosphopeptide and fluoride on the remineralization: *in vitro* study

**DOI:** 10.1590/1807-3107bor-2024.vol38.0036

**Published:** 2024-05-13

**Authors:** Marília Andrade Figueiredo de OLIVEIRA, Francyenne Maira Castro GONÇALVES, Alberto Carlos Botazzo DELBEM, Gabriela Leal Peres FERNANDES, Mark L. CANNON, Marcelle DANELON

**Affiliations:** (a) Universidade de Ribeirão Preto – Unaerp, School of Dentistry, Ribeirão Preto, SP – Brazil.; (b) Universidade Estadual Paulista – Unesp, School of Dentistry, Department of Preventive and Restorative Dentistry, Araçatuba, SP, Brazil.; (c) Northwestern University, Feinberg School of Medicine, Ann and Robert Lurie Children’s Hospital, Chicago, IL, USA.

**Keywords:** Dental Enamel, Fluorides, Phosphates, Hardness, Caseins

## Abstract

This study aimed to evaluate *in vitro* the effect protocols and anticaries agents containing casein amorphous calcium fluoride phosphopeptide-phosphate (CPP-ACPF, MI Paste Plus), sodium trimetaphosphate (TMP) and fluoride (F), in remineralization of caries lesions. Bovine enamel blocks with initial caries lesions were divided into groups (n = 12): 1) Toothpaste without F-TMP-MI Plus (Placebo); 2) Toothpaste 1100 ppm F (1100F), 3) 1100F + MI Paste Plus (1100F-MI Paste Plus), 4) Toothpaste with 1100F + Neutral gel with 4,500 ppm F + 5%TMP (1100F + Gel TMP) and 5) Toothpaste with 1100F + Neutral gel with 9,000 ppm F (1100F + Gel F). For the 4 and 5 groups the gel was applied only once for 1 minute, initially to the study. For the 3 group, after treatment with 1100F, MI Paste Plus was applied 2x/day for 3 minute. After pH cycling, the percentage of surface hardness recovery (%SH_R_); integrated loss of subsurface hardness (ΔKHN); profile and depth of the subsuperficial lesion (PLM); concentrations of F, calcium (Ca) and phosphorus (P) in enamel was determined. The data were analyzed by ANOVA (1-criterion) and Student-Newman-Keuls test (p < 0.001). Treatment with 1100F alone led to ~ 28% higher remineralization when compared to treatment with 1100F associated with MI Paste Plus (p < 0.001). The 1100F and 1100F + Gel F groups showed similar values for %SH_R_ (p = 0.150). 1100F + Gel TMP treatment also remineralized the enamel surface by ~ 30% and 20% when compared to the 1100F + Gel F and 1100F groups (p < 0.001). The lower lesion depth (ΔKHN) was observed for the 1100F + Gel TMP group (p < 0.001), where it was 54% and 44% lower in comparison to the 1100F and 1100F + Gel F groups (p < 0.001). Polarized light microscopy photomicrographs showed subsurface lesions in all groups, but these lesions were present to a lower extent in the 1100F + Gel TMP group (p < 0.001). Treatment with 1100F + Gel TMP promoted an increase in the concentration of Ca in the enamel by ~ 57% and ~ 26% when compared to the 1100F and 1100F + MI Paste Plus groups (p < 0.001), respectively. There were no significant differences between the 1100F, 1100F + MI Paste Plus and 1100F + Gel F groups (p > 0.001). Similar values of P in the enamel were observed in the 1100F, 1100F + MI Paste Plus and 1100F + Gel F groups (p > 0.001), except for the 1100F + Gel TMP group, which presented a high concentration (p < 0.001). We conclude that the 1100F+TMP gel treatment/protocol led to a significant increased remineralization when compared to the other treatments/protocols and may be a promising strategy for patients with early caries lesions.

## Introduction

Dental caries is still considered a public health problem, affecting about 35% of humans worldwide, especially in childhood, when the disease is most prevalent.^1^. Through the biofilm, articulated by cariogenic diet, microbiota, and inadequate hygiene, dental caries has a complex and multifactorial etiology, and has been considered a biofilm-dependent disease.^2^

In order to preserve tooth structure, fluoride (F) is recommended as a preventive and remineralizing agent for initial caries lesions.^3^ Fluoride vehicles are arranged as varnishes, gels, mouthwashes, and toothpastes. When a product with F concentration is applied on the tooth surface, there is deposition of calcium fluoride (CaF_2_), which is covered by calcium (Ca) and phosphate (P) ions, and saliva proteins that delay mineral solubility.^4^ Thus, it functions as a source of F, thereby interfering with the dynamics of the de-remineralization processes.^5^ To maximize the clinical significance of remineralization, a number of preventive agents in some studies have shown successes in using P, Ca ions associated with F on the tooth surface, assisting in enamel remineralization.^3,6,7^

The performance of dairy products, as a topical anticariogenic effect, has been noted since the 1980s.This protective effect is due to casein phosphoprotein and calcium phosphate,^8,9^ as these are rich sources of Ca and P ions, that are available to the biofilm, thus reducing tooth demineralization. The casein phosphopeptide amorphous calcium phosphate (CPP-ACP) complex links to and utilizes casein phosphopeptide (CPP) to stabilize amorphous calcium phosphate (ACP).^10^ The addition of CPP to the acquired salivary pellicle decreases S. mutans adherence significantly. Furthermore, the high concentrations of extracellular free calcium dispensed by CPP-ACP complexes may increase the permeability of the streptococcus membrane and promote partial lysis. In addition, CCP-ACP has the ability to bind to plaque, providing a considerable calcium reservoir within the plaque and reduces the diffusion of free calcium. In return, this restricts mineral loss during a cariogenic loss during a cariogenic process and provides a possible source of calcium for remineralization.^11^ When associated with 900 ppm F^-^, casein phosphopeptide-amorphous calcium fluoride phosphate (CPP-ACPF) becomes commercially available for professional use as a paste (MI Paste Plus). According to some studies, this addition of F increases the remineralizing effect of initial caries lesions when compared to CPP-ACP alone.^12,13^ This occurs due to the additional and distinct anticariogenic effect of CPP-ACP acting to prevent demineralization and F, forming fluorapatite, promoting remineralization and reducing tooth demineralization.^12^

In addition to CPP-ACPF, Sodium Trimetaphosphate (TMP), a condensed cyclic phosphate, with a high cariostatic potential and when added to toothpastes, gels, varnishes and mouthwashes showed a reduction in enamel demineralization and an increase in remineralization.^14-19^ This effect is attributed to the behavior of TMP under acid challenge, which has been shown to reduce mineral loss. It also increases the remineralization process, due to its ability to modify the tooth surface and to promote the diffusion of ions inside the tooth, contributing to a greater incorporation of Ca, P, and F ions.^9,17,18^ In recent years, some studies have evaluated the effects of the association of TMP (in a 5% concentration) in F gels with 4,500 ppm F on the demineralization and remineralization processes, evidencing that it is possible to reduce the amount of F in the product, keeping the efficacy similar to the acidulated gel with 12,300 ppm F and superior the gel with 9,000 ppm F,^14,16,20^ therefore the use of products with low-fluoride concentration may permit the use of gels in children with greater security and the addition of phosphates it is possible and giving a great benefice.

Nevertheless, despite the numerous sources of F, there are populations of infants that concentrate a high prevalence of dental caries. Considering the ability of TMP to reduce acid diffusion and CPP-ACPF as a source of Ca, P, and F this study aimed to evaluate *in vitro* the effect protocols and anticaries agents containing casein amorphous calcium fluoride phosphopeptide-phosphate (CPP-ACPF, MI Paste Plus), sodium trimetaphosphate (TMP) and fluoride (F), in remineralization of caries lesions. The null hypothesis investigated was that treatment with 1100 ppm F + gel containing 4,500 ppm F + 5%TMP would not increase remineralization of initial caries lesions when compared to treatment with 1100 ppm F + MI Paste Plus, not influenced by the treatment protocol.

## Methodology

### Experimental design

Bovine enamel blocks (4 mm × 4 mm, n = 60) were selected for their initial surface hardness (SH). They were then subjected to the induction of artificial caries lesions, and the post-demineralization hardness (SH_1_) was determined. Next, bovine enamel blocks were randomly allocated into six groups (n = 12), according to the treatments: a) Toothpaste without F-TMP-MI Paste Plus (Placebo); b) Toothpaste 1100 ppm F (1100F), c) 1100F + MI Paste Plus (1100F-MI Paste Plus), d) Toothpaste with 1100 ppm F + Neutral gel with 4,500 ppm F + 5%TMP (1100F + Gel TMP) and e) Toothpaste with 1100 ppm F + Neutral gel with 9,000 ppm F (1100F + Gel F). The 9,000 ppm F gel was selected for the study given based on significant evidence in caries control and application in clinical practice. Regarding the concentration of 4500 ppm F associated with 5%TMP, it was based on the previous studies.^14,16^ The sample size of 12 enamel blocks was based on a pilot study, adopting the surface and cross-sectional hardness as the primary outcomes, the mean difference between groups (10 and 2800, respectively), standard deviation (4 and 1500, respectively), an α-error of 5%, and a β-error of 10%. The blocks were treated twice daily with slurries of toothpastes (1 minute) and, in addition, group 3 received the application of MI Paste Plus for 3 minutes. For groups 4 and 5, the gel was applied only once for 1 minute, before being submitted to pH cycling. After pH cycling (demineralizing and remineralizing solution), the following were determined: percentage of surface hardness recovery (%SH_R_); integrated loss of subsurface hardness (ΔKHN); profile analysis and lesion depth subsurface through polarized light microscopy (PLM); fluoride (F), calcium (Ca) and phosphorus (P) concentrations in the enamel.

### Formulation, pH determination and F concentration in experimental toothpastes/gels and MI Paste Plus

The experimental toothpastes were produced with the following components: titanium dioxide (Sigma-Aldrich); carboxymethyl cellulose (Sigma-Aldrich); methyl p-hydroxybenzoate sodium (Sigma-Aldrich); saccharin (Vetec); peppermint oil (Synth); glycerol (Sigma-Aldrich); abrasive silica (Tixosil 73); and sodium lauryl sulfate (Sigma-Aldrich), adjusted with deionized water to 100 g. NaF (Merck), was added to the F toothpaste to reach a concentration of 1,100 ppm F.^21,22^ . A toothpaste with no added F (Placebo) was prepared using the same formulation as the others, and a commercial paste containing CPP-ACP associated with 900 ppm F-MI Paste Plus was used (MI Paste Plus). Before the study, total fluoride (TF)/ionic fluoride (IF) concentrations and the pH of the toothpastes/MI Paste Plus, were all verified using an ion-specific electrode (9409 BN) connected with an ion analyzer (Orion 720), previously calibrated with 5 standards (0.125, 0.25, 0.5, 1.0 and 2.0 mg F/mL).

An experimental gel with neutral pH was prepared in the laboratory with the following ingredients: carboxymethyl cellulose (Synth), sodium saccharin (Vetec), glycerol (Sigma-Aldrich) and peppermint oil (Synth) adjusted with deionized water to 100 g. F (NaF, Merck) was added to the gel at concentrations of 4,500 or 9,000 ppm F. Subsequently, TMP (Sigma-Aldrich) was added at a 5% concentration to the gel with F concentrations of 4,500 ppm F.

The F concentrations in the toothpastes, gels and MI Paste Plus were determined with a F ion specific electrode (9609 BN) attached to an ion analyzer (Orion 720 A+) and calibrated with standards containing 0.125–2.000 ppm F. The pH levels of the gels and toothpaste were determined with a pH electrode (2A09E, Analyser) that was calibrated with standard pH levels of 7.0 and 4.0.^14,22^

### Induction of artificial caries lesions in enamel blocks

For the induction of artificial caries lesions, all surfaces of each specimen, except the enamel surface, were coated with acid-resistant varnish and placed individually in a demineralizing solution (1.3 mmol. L^-1^ Ca; 0.78 mmol.L^-1^ P in 0.05 mol/L acetate buffer, at pH 5.0; 0.03 ppm F; 32 mL/block) for a period of 16 h at 37^o^C. Thereafter, the post-demineralization surface hardness (SH_1_) was determined.^9^

### pH cycling (RE > DES) and treatment

The blocks were subjected to pH cycling in individual vials for 6 days at 37°C. The blocks were treated twice daily with slurries of toothpastes (1 minute);^23^ in addition, group 3 after treatment with slurry of toothpaste (1100F), received the application of MI Paste Plus for 3 minutes (according to the manufacturer’s instructions), and then the blocks were washed with deionized water. For groups 4 and 5, after treatment with slurry of toothpaste (1100F), the gel was applied only once for 1 minute,^14,16,20^ and then the blocks were washed with deionized water. Immediately, the blocks (n = 60) were immersed in the remineralizing solution (RE - Ca 1.5 mmol L^-1^Ca(NO_3_)_2_.4H_2_O, P 0.9 mmol L^-1^, NaH_2_PO_4_.H_2_O, KCl 0.15 mol L^-1^, in 0.02 mol cacodylate buffer L^-1^, 0.05 µg F/mL at pH 7.0, 1.1 mL/mm^2^) which was changed twice a day (8 a.m and 4 p.m); then, they were immersed in the demineralizing solution (DES - Ca 2.0 mmol L^-1^ Ca(NO_3_)_2_.4H_2_O and P 2.0 mmol L^-1^ NaH_2_PO_4_.H_2_O in 0.075 mol L^-1^ acetate buffer, 0.04 µg F/mL at pH 4.7, 2.2 mL/mm^2^) for 2 hours (12 p.m to 2 p.m)^8^. The blocks were washed with jets of deionized water for 30 seconds, after being removed from the DES-RE solutions, and then dried with absorbent paper. After 6 days of pH cycling, the post-cycling surface hardness (SH_2_) was determined to calculate the percentage of surface hardness recovery (%SH_R_).^9^

### Analysis of enamel hardness

The surface hardness was determined using the Micromet 5114 hardness tester (Buehler) and the Buehler Omni Met software (Buehler), with a Knoop diamond indenter under a 25 g load for 10 s. Five impressions, separated by a distance of 100 μm, were made in the central region of each block, for the analysis of the initial surface hardness (SH). After the induction of artificial caries lesions, another five impressions (SH_1_) were made at 100 μm from the SH impressions. After pH cycling, another five impressions were made for the analysis of the final hardness (SH_2_) at 100 µm from the SH_1_ impressions, and then we calculated the percentage of surface hardness recovery (%SH_R_). For hardness measurements, a section was made in the center of each block, and one of the halves was embedded in acrylic resin and gradually polished. One sequence of 14 indentations was created at different distances (5, 10, 15, 20, 25, 30, 40, 50, 70, 90, 110, 130, 220, and 330 μm) from the surface of the enamel, in the central region of the blocks, using a Micromet 5114 hardness tester (Buehler ) with a Knoop diamond indenter under a 5 g load for 10 s. Integrated hardness (KHN × μm) for the lesion into sound enamel was calculated by the trapezoidal rule (GraphPad Prism, version 3.02) and subtracted from the integrated hardness for sound enamel to obtain the integrated area of the subsurface regions in the enamel, obtaining the integrated loss of subsurface hardness (∆KHN; KHN × μm).^9,22^

### Analysis of the profile and depth of subsurface lesions using polarized light microscopy

After cross-sectional hardness analysis, the enamel blocks (n = 12/group) embedded in acrilyc resin were sectioned to obtain slices of 300 µm and ground to a thickness of ~100 um using 400 grit paper (Paper Discs,30-5108-320, Buehler) at grinder polisher (Phoenix Beta with Vector Powerhead, Buehler), under constant water refrigeration. Then, the enamel slices were manually polished in a sequence of sandpaper (600, 800, and 1200 grit sandpaper, Buehler) and deionized water, and mounted on glass slides with deionized water and covered with a coverslip glass, the edges of which were sealed with synthetic resin (Entellan, Merck). The presence and thickness (µm) of surface layer of the enamel and depth of artificial demineralization (µm) were measured at three areas from the central region of the slices at ×40 magnification in polarized light microscopy (PLM).^9,16^

### Analysis of the F, Ca and P concentration in enamel

One of the halves of the longitudinally sectioned blocks was sectioned again in order to obtain blocks with a thickness of 2 mm x 2 mm and then fixed with adhesive glue in a mandrel for a straight piece. A digital caliper (Mitutoyo CD-15B) was used to measure the surface area of the enamel blocks. Next, the blocks were fixed to a mandrel coupled to a modified microscope with a micrometer (Micrometer 733 MEXFLZ-50, Starret) to measure enamel wear. Self-adhesive polishing disc (13 mm diameter) and 400-grit silicon carbide (Buelher) were fixed in crystal polystyrene flasks (J-10). One layer of enamel (~ 50 µm) was removed from each block.^24^ Then, 0.5 mL of 0.5 mol L^-1^ HCl was added to the resulting enamel powder retained on the polishing discs; this mixture was then agitated for 1 h. For F analysis, specific electrode 9409BN and microelectrode reference (Analyser), coupled to an ion analyzer (Orion 720A+), was utilized. The electrodes were calibrated with standards ranging from 0.25 to 4.00 ppm of F (100 ppm F, Orion 940,907), under the same conditions as the samples. The readings were conducted in 0.300 mL of the biopsy solution with the same volume of TISAB II modified NaOH.^20,25^ The data obtained in mV were converted to μg/mm^3^ using Excel spreadsheet.

Ca analysis was performed using the Arsenazo III colorimetric method as described by Vogel et al.^25^ The absorbance readings were recorded at 650 nm with a plate reader (PowerWave 340). P was measured according to Fiske and Subbarow,^26^ and the absorbance readings were recorded at 660 nm. The results were expressed as μg/mm^3^.

### Statistical analysis

The analysis was performed using the SigmaPlot software (version 12.0, Systat Software), at a significance level of 5%. The variables %SH_R_, ∆KHN, PLM, F, Ca and P exhibited normal (Shapiro-Wilk test) and homogeneous (Cochran test) distributions and, thereafter, were analyzed by ANOVA 1-criterion, followed by the Student-Newman-Keuls test.

## Results

Mean (SD) of total fluoride (TF) and ionic fluoride (IF) concentrations (ppm F), respectively, were 10.5 (0.1) and 10.0 (1.2) in the Placebo, 1186.0 (33.2) and 1102.4 (28.5) in the 1100F, 913.3 (18.4) and 900.3 (24.7) in the MI Paste Plus. IF concentrations (ppm F) in the Gel TMP and Gel F were, respectively: 4,502.8 (11.3) and 9,050.0 (12.7). The mean pH value of the toothpastes and MI Paste Plus was 7.4 (0.1) ranging from 7.3 to 7.5. The pH of the neutral gels was 7.2 (0.2), ranging from 7.2 to 7.3.

**Figure 1:**
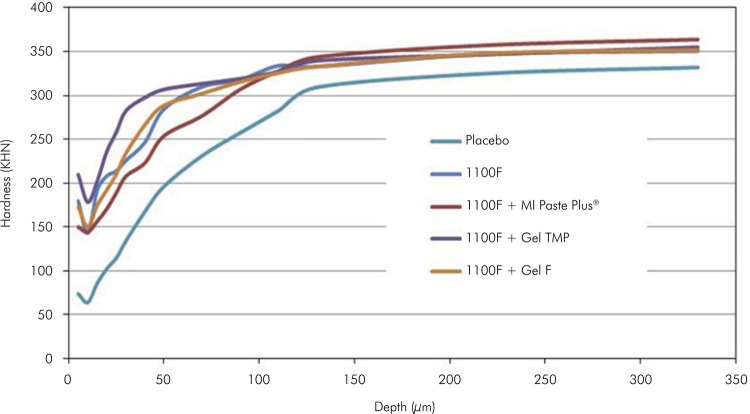
Cross-sectional hardness profiles (mean KHN, n = 12) at different depths (micrometers) in enamel blocks according to the treatments.

**Figure 2:**
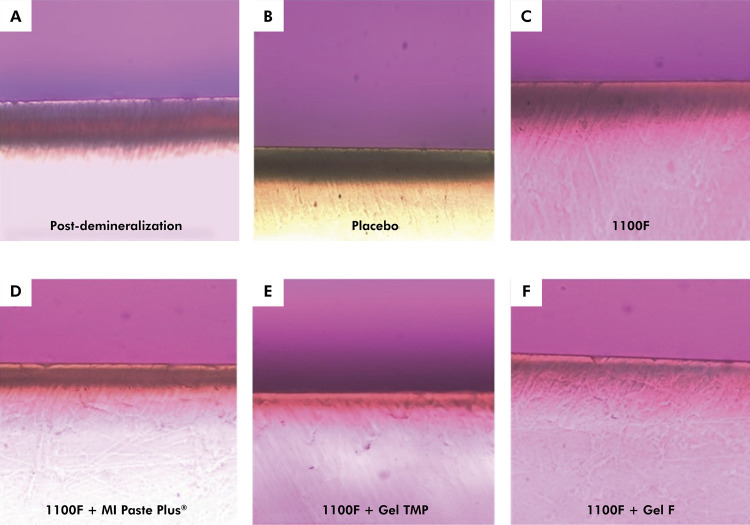
Photomicrograph with polarized light of the lesion formed before and after treatments: a) post-demineralization; (b) Placebo; (c) 1100F; (d) 1100F + MI Paste Plus®; (e) 1100F + Gel TMP; (f) 1100F + Gel F (× 40).

The mean (SD) surface hardness (SH) for all blocks was 366.6 (2.1) KHN, ranging from 363.2 (5.2) to 366.7 (3.9) in the experimental groups and there was no statistical difference between them (p = 0.150). The mean (SD) of post-demineralization surface hardness (SH_1_) was 58.4 (5.2) KHN, ranging from 50.4 to 64.6 (p = 0.093). There were no significant differences between groups after distribution (p = 0.077). Placebo group showed the lowest values for %SH_R_ (p > 0.001). Treatment with 1100F alone led to ~ 28% higher remineralization when compared to treatment with 1100F associated with MI Paste Plus (p < 0.001). The 1100F and 1100F + Gel F groups showed similar values (p = 0.150). 1100F + Gel TMP treatment also remineralized the enamel surface by ~ 30% and 20% when compared to the 1100F + Gel F and 1100F groups (p < 0.001) (Table 1). The lower lesion depth (ΔKHN) was observed for the 1100F + Gel TMP group (p < 0.001), where it was 54% and 44% lower in comparison to the 1100F and 1100F + Gel F groups (p < 0.001) (Table 1, Figure 1). Polarized light microscopy photomicrographs showed subsurface lesions in all groups, but these lesions were present to a lower extent in the 1100F + Gel TMP group (Table 1, Figure 2).

**Table 1:** Mean (± SD) of the variables analyzed in the enamel according to the treatments.

Treatments	%SH_R_		ΔKHN		PLM Superficial		PLM Depth	

	(KHN)	± SD	(KHN × µm)	± SD	µm	± SD	µm	± SD
Post-demineralization			9,956.5^e^	(1,279.2)	0.3^a^	(0.1)	37.9^c^	(1.4)
Placebo	14.8^a^	(3.0)	7,451.0^d^	(1,112.4)	1.5^a^	(0.3)	27.9^c^	(5.3)
1100F	36.4^c^	(6.1)	3,513.9^c^	(710.6)	3.1^b^	(0.4)	17.4^b^	(4.3)
1100F + MI Paste Plus	28.4^b^	(3.8)	4,769.6^c^	(950.5)	2.7^b^	(0.8)	12.6^a^	(2.4)
1100F + Gel TMP	43.9^d^	(3.9)	1,625.4^a^	(858.3)	3.7^c^	(0.6)	12.4^a^	(6.2)
1100F + Gel F	34.0^c^	(3.9)	2,921.5^b^	(632.4)	2.1^b^	(1.0)	11.4^a^	(2.6)

Distinct superscript letters indicate statistical significance among the treatment in each analysis (One-way ANOVA, followed Student–Newman–Keuls’ test). Values represent means (standard deviations). %SH_R_: percentage of surface hardness recovery. ΔKHN: integrated subsurface hardness loss. PLM surface: thickness of the enamel surface layer. PLM depth: thickness of demineralization depth. KHN: Knoop hardness. μm: micrometers.

The concentration values of F, Ca and P in the enamel are presented in Table 2. The concentration of F was similar between the fluoride groups (p = 1.789). The 1100F + Gel TMP group showed the highest values of Ca in the enamel with an increase of ~ 57% and ~ 26% when compared to the 1100F and 1100F + MI Paste Plus groups (p < 0.001), respectively. There were no significant differences between the 1100F, 1100F + MI Paste Plus and 1100F + Gel F groups (p > 0.001) (Table 2). Similar values of P in the enamel were observed in the 1100F, 1100F + MI Paste Plus and 1100F + Gel F groups (p > 0.001), except for the 1100F + Gel TMP group, which presented a high concentration (p < 0.001).

**Table 2:** Mean (± SD) of F, Ca and P in enamel according to treatments.

Treatments	F		Ca		P	

	(µg/mm^3^)	± SD	(µg/mm^3^)	± SD	(µg/mm^3^)	± SD
Placebo	1.7^a^	(0.1)	277.8^a^	(50.7)	296.4^a^	(44.2)
1100F	2.7^b^	(1.0)	501.0^b^	(85.2)	372.2^b^	(46.0)
1100F + MI Paste Plus	2.3^b^	(0.6)	626.1^b^	(173.9)	433.9^c^	(56.9)
1100F + Gel TMP	3.2^b^	(0.8)	785.6^c^	(108.9)	519.6^d^	(84.2)
1100F + Gel F	2.6^b^	(0.8)	570.7^b^	(190.1)	388.3^b^	(60.3)

Distinct superscript letters indicate statistical significance among the treatments in each analysis (One-way ANOVA, followed Student–Newman–Keuls’ test). Values represent means (standard deviations). F: fluoride in the enamel. Ca: calcium in the enamel. P: phosphorus in the enamel.

## Discussion

Different therapies in caries control have been proposed in the literature, among which we highlight the use of F in association with Ca and P, as the presence of these ions in the salivary environment and biofilm positively interfere in the processes of dental remineralization and demineralization.^8,9,22^ The null hypothesis investigated was that treatment with 1100 ppm F + gel containing 4,500 ppm F + 5%TMP would not increase remineralization of initial caries lesions when compared to treatment with 1100 ppm F + MI Paste Plus, not influenced by the treatment protocol. Given the results obtained, the null hypothesis was rejected. Although there are numerous formulations and treatment protocols, with different active agents on dental remineralization, contradictory results are observed in the literature regarding the formulations when applied to initial caries lesions. In this sense, it is interesting to evaluate remineralizing agents containing the casein calcium phosphate amorphous fluoride phosphopeptide complex (ACP-CPPF), sodium trimetaphosphate (TMP) and fluoride (F) alone, as well as the treatment protocols of these agents, to obtain more evidence about their effect on the remineralization of initial caries lesions, since they are being used as a preventive strategy to reduce dental caries, especially for patients with high experience of the disease.

Previous studies have demonstrated that CPP-ACP exhibits good ability to penetrate and remineralized the lesion body when compared to other remineralizing agents.^8,12,27^ It is suggested that the Ca and P released by CPP-ACP have the capability to form nano-complexes in the biofilm adjacent to the initial caries lesion, increasing enamel resistance to acidic challenges.^28^ Also, when F is associated with CPP-ACP (CPP-ACPF), it provides superior effects on remineralization and greater resistance to tooth demineralization.^29^ In situations of high caries risk, the combination of topical methods can be as suggested; however, not always saturating the environment with these ions can contribute to a synergistic effect, that is, the combination should take into account the mechanisms of action, preventing competition for specific sites in the dental structure, which could lead to an antagonistic effect.^30^

During the development of this study, it was not possible to find in the literature studies associating the use of conventional toothpaste (1100F) with MI Paste Plus on the remineralization of initial caries lesions, which is of great significance, since it would simulate the daily clinical condition of an individual. Meyer-Lueckel et al.^27^ evaluated in an in situ study the remineralizing effects induced by the application of MI Paste, without F, after the use of F toothpaste (1,400 ppm F), on the remineralization of caries lesions in enamel. The authors concluded that the additional use of a CPP-ACP paste seems to be less effective in remineralizing caries lesions than the continuous use with fluoride toothpaste, supporting the hypothesis of the occurrence of an antagonistic rather than synergistic effect between the active agents. These results are also reported by other authors.^9,29^ It is important to emphasize that many protocols that use MI Paste Plus for the treatment of caries lesions, do not associate the treatment with conventional toothpaste, and in this way the ideal clinical condition of brushing is not simulated. Another important aspect is that MI Paste Plus is not a toothpaste that should be diluted and applied during brushing; according to the manufacturer, it should be applied after the patient’s oral hygiene, using the finger in the affected dental areas. Our findings showed that the association of 1100F + MI Paste Plus did not increase the remineralization of lesions both on the surface and in depth, evidencing that this association may worsen the effect of daily 1100F toothpaste (Table 2, Figure 1 and Figure 2).

For this finding, we would suggest initially that pH cycling models do not simulate the oral environment precisely, which can directly influence the ion exchange of the environment and the tooth surface; another important consideration is the absence of biofilm and, consequently, a more favorable environment for ACP-ACPF to create a state of supersaturation of Ca and P ions, which may be preventing this system from adequately exerting its remineralizing action; in addition to the treatment period^31^. Our results are in agreement with those of Gonçalves et al.^9^ and Souza et al.^32^ suggesting that a chemical reaction may occur between ACP and F, making the two inorganic components ineffective. Another hypothesis is that increased Ca and P ions release on the demineralized enamel surface may have occurred and caused greater precipitation of these on the surface, especially when associated with F ions^33^. It was suggested that, by the precipitation of these ions occurring rapidly in the superficial zones of the lesion, there is impediment to the remineralization process to occur in the body of the lesion, as demonstrated in our study, since the association of these treatments (1100F + MI Paste Plus). Even though it contributed to a higher Ca ions concentration (approximately 25%) in the enamel when compared to the 1100F toothpaste, their capacity to remineralize the enamel subsurface lesion was lower (Table 1, Table 2, Figure 1 and Figure 2). It is possible that enamel pore obstruction occurs, impeding the diffusion of neutral species (HF^0^ and CaHPO_4_^0^) into the lesion body, consequently reducing remineralization^30,33^.

Thus, in order to avoid mineral precipitation on the tooth surface produced by calcium and phosphate containing systems^30,33^ and to allow biomineralization deep into the lesion, adsorption of cyclophosphate (e.g.: TMP) onto enamel has been shown to be a promising strategy, as it keeps the pores open and facilitates ion diffusion into the enamel^9,21,34^. The use of TMP concentration of 5% was based on the study by Danelon et al.^14^, where the authors demonstrated an in situ remineralization study that the supplementation of a gel with 4,500 ppm F led to a remineralizing effect similar to gels with 9,000 and Acid Gel (12,300 ppm F). Furthermore, Akabane et al.,^20^evaluated in situ the same gel (4500 ppm F + 5%TMP) in association with conventional F toothpaste (1100 ppm F) on enamel demineralization and biofilm, proving that this association reduces enamel demineralization on surface and in depth and changes the composition of the biofilm (reducing the production of extracellular polysaccharides). It can thus be an alternative treatment for patients with high caries risk, without the need to use gels of conventional concentration. In our study, the association of 1100F + TMP Gel led to greater enamel remineralization by ~ 30% and 20% when compared to the 1100F + Gel F and 1100F groups, respectively (Table 2). In addition, the lower lesion depth (ΔKHN and MLP data) was observed for the 1100F + Gel TMP group, being 54% and 44% when compared to the 1100F and 1100F + Gel F groups (Table 2, Figure 1 and Figure 2). It is important to highlight that, according to the data in Table 2, TMP facilitated the diffusion of P and Ca ions, but not of F. According to these data, it is believed that it runs the formation of an apatite-like layer of calcium phosphate^34^ in the presence of TMP, since this cyclophosphate works as a biomimetic material that induces apatite-like crystal deposits in the enamel structure occurring repair, as demonstrated in other studies^9,35^. The %SH_R_ data of the 1100F + MI Paste Plus group (Table 2) support the hypothesis that direct precipitation in enamel leads to pore obliteration, reducing remineralization capacity.

When a remineralizing agent is used, it is expected that its action will take effect in a short period of time. Therefore, important variables should be considered before determining the experimental period of an *in vitro* study, such as the type of substrate and depth of caries lesions. Bovine enamel has a higher reactivity and porosity, leading to a remineralization more rapidly when compared to human enamel^35^. In addition, as for the substrate, the depth of enamel demineralization may also interfere with remineralization according to time; however, few investigations have considered the depth of the demineralized area in their protocols. The potential to demineralize the lesion at the subsurface is of fundamental importance, since within the body of the lesion a loss of up to 50% of original mineral content can occur and is often covered by an apparently intact surface layer^31^. The caries lesion formed in this *in vitro* model is superficial, and the 6-day protocol promoted by pH cycling was sufficient to evaluate the differences between the groups, confirming data from studies that demonstrated how this *in vitro* model was able to verify response and dose when agents with different concentrations of fluoride were used.^9,16,21^ In addition, according to Lynch et al.,^37^ these lesions promote better discrimination and ability to verify efficacy between treatments.

It is important to emphasize that many protocols that use MI Paste Plus for the treatment of caries lesions do not associate it with the treatment with conventional toothpaste, and thus do not simulate the ideal clinical condition of brushing. Another important aspect is that MI Paste Plus is not a toothpaste that should be diluted and applied during brushing; according to the manufacturer, it should be applied after the patient’s oral hygiene, using the finger on the affected tooth areas, which differs from the application of toothpaste and gel used in our study. Still, *in vitro* studies cannot simulate all the complexities of an oral cariogenic environment in vivo. Therefore, the results obtained in this study are the initial evidence to encourage further exploration of the philosophy of multiple mechanisms that act together for caries prevention. Some examples of future studies that should be conducted include: a) amount of product retained on enamel after mouth rinsing; b) presence of human saliva; c) different protocols (application time x amount/day) and d) presence of biofilm and, consequently, a favorable environment for CPP-ACPF to contribute to a state of supersaturation of Ca and P ions, through in situ and in vivo studies. We conclude that the 1100F+TMP gel treatment/protocol led to a significant increased remineralization when compared to the other treatments/protocols and may be a promising strategy for patients with initial caries lesions.
